# Is duration of psychological treatment for depression related to return into treatment?

**DOI:** 10.1007/s00127-016-1267-7

**Published:** 2016-07-23

**Authors:** A. M. Boerema, P. Cuijpers, A. T. F. Beekman, A. Hellenthal, L. Voorrips, A. van Straten

**Affiliations:** 1 Department of Clinical Neuro and Developmental Psychology, Section Clinical Psychology, Faculty of Behavioural and Movement Sciences, Vrije Universiteit Amsterdam, van der Boechorststraat 1, 1081 BT Amsterdam, The Netherlands; 2 EMGO+ Institute for Health Care and Research, VU University Medical Centre, van der Boechorststraat 7, 1081 BT Amsterdam, The Netherlands; 3 Department of Psychiatry, VU University Medical Centre, van der Boechorststraat 7, 1081 BT Amsterdam, The Netherlands; 4 Statistics Netherlands (CBS), Henri Faasdreef 312, 2492 JP The Hague, The Netherlands

**Keywords:** Duration of treatment, Return into treatment, Brief therapy, Psychotherapy

## Abstract

**Purpose:**

There is increasing pressure on mental health providers to reduce the duration of treatments, while retaining level of quality and effectiveness. The risk is that the population is underserved and therefore needs new treatment episodes. The primary aim of this study was to investigate whether duration of treatment and return into mental health care were related.

**Methods:**

This study examined Dutch patients with an initial treatment episode in 2009 or 2010 in specialized mental health settings for depressive disorder (*N* = 85,754). Follow-up data about treatment episodes were available up until 2013. The data set included demographic (age, gender), and clinical factors (comorbidity with other DSM-IV Axis; scores on the ‘Global Assessment of Functioning’). Cox regression analyses were used to assess whether duration of treatment and relapse into mental health care were related.

**Results:**

The majority of patients did not return into mental health care (86 %). Patients with a shorter duration of treatment (5–250 min; 251–500 min and 751–1000 min) were slightly more likely to return (reference group: >1000 min) (HR 1.19 95 % CI 1.13–1.26; HR 1.11 95 % CI 1.06–1.17; HR 1.18 95 % CI 1.11–1.25), adjusted for demographic and clinical variables.

**Conclusions:**

The results suggest that a longer duration of treatment may prevent return into mental health care in some groups. However, because of the design of the study, no causal inference can be drawn. Further research, preferably in a RCT, is needed to determine whether the trend towards lower intensity treatments is associated with repeated mental health care use.

## Introduction

In the past decades, psychotherapy for depression shifted from long-term psycho-analytic approaches to more brief therapies like cognitive behavioral therapy [1]. At present there is an increasing pressure on mental health-care providers to provide even shorter therapies, while retaining high quality and cost-effectiveness [1]. These treatments typically focus on increasing patients’ self-management skills and may be offered face-to-face, by telephone or online. There is evidence that such brief therapies are effective [2–7]. Furthermore, many guidelines recommend low intensity treatment as a first step in the treatment of mild to moderate depression [8–10] and provide more intensive treatment only to people who do not respond to low intensity treatments [11]. Research demonstrated that this stepped approach is equally effective when compared to the traditional ‘matched care’ (in which a multidisciplinary team matches client and therapy) [12]. A recent meta-regression analysis demonstrated, in addition, that duration of treatment is not associated with therapeutic effectiveness, suggesting that brief treatments may have equal outcomes but could be more cost effective [3]. In contrast with this, some studies showed that a longer duration of treatment may be more beneficial, due to the continued improvement of patients during therapy [13]. Moreover, there is evidence that treatment duration in mental health practice is associated with patients characteristics [14]. Patients with more severe and complex symptoms receive treatments with a longer duration [15]. On the other hand, complexity of the disorder is also associated with a higher risk for drop out [16, 17], implying that people with more severe symptoms possibly receive a shorter duration of therapy even though there was no intention to offer only brief therapy. A concern with brief therapies is that patients might improve but perhaps not fully recover [13]. Since residual symptoms are strongly associated with recurrence [18, 19]; this might lead to repeated mental health care use.

Repeated mental health care use is a relatively common phenomenon. Findings in the Netherlands show that almost a quarter of the people who started a treatment in 2006 for psychological problems received treatment a second time and 16 % entered treatment three times or more [20]. It is also well known that depression is a condition with a high risk of recurrence [18, 21] leading to repeated mental health care use. Although there is considerable attention in research on establishing treatments that reduce the risk of recurrence in depression [22–25], there is to the best of our knowledge no information available on the association between the duration of the initial treatment and the risk of return into mental health care. Gaining insight in this association might provide better understanding of the long-term outcomes of brief therapies. Furthermore, examining how treatment guidelines are implemented in practice (e.g., how many people actually receive a brief or prolonged therapy) could provide useful information of clinical practice in people with depression.

The aim of this paper examines the association between the duration of initial treatment and the risk of return into mental health care treatment in a large naturalistic cohort of patients treated for depression in specialized mental health care. All patients receiving outpatient treatment in 2009 or 2010 in the Netherlands were included and follow-up data were available for 3 years. In addition, we examined the association between duration of initial treatment and the risk of return into mental health care for four treatment groups as well (e.g., patients who received psychotherapy only, medication only, psychotherapy and medication, other therapy). Furthermore, we examined which demographic (age, gender) and clinical factors (severity of symptoms) contribute to the risk of returning into mental health care.

## Methods

### Data

Data collected in routine practice were available from mental health care providers in the Netherlands. Mental health practitioners routinely register diagnoses and treatments in Diagnosis Treatment Combinations (DTCs) [26]. The DTC contains information about the type of care (e.g., regular care or crisis), the diagnosis (DSM-IV classification) and the received treatment (e.g., psychotherapy and/or pharmacotherapy). With the DTC system, mental health care providers are able to register their delivered care in a systematic way. Furthermore, the DTC information is used by health insurance companies as a national and compulsory basis for reimbursement of the delivered treatment in mental health care settings (e.g., a reimbursement system). Furthermore, all mental health care providers are obliged to provide their anonymized DTC information to the DIS system (DTC Information System) which receives, gathers and safely stores DTC information.

The data for the present study were used in collaboration with Statistics Netherlands (CBS). CBS has the legal right by law to process this type of information from the DIS system, in order to publish official national statistics about specialized mental health care in the Netherlands. The data included only information about the registered treatment activities and was not traceable to individual persons. In the present study, data were used from the period between 2009 and 2013.

### Sample

Patients were included in the present study if they were diagnosed with a depressive disorder (based on DSM-IV-TR criteria), started a treatment in 2009 or 2010, received outpatient treatment and were 18 years or older.

**Fig. 1:**
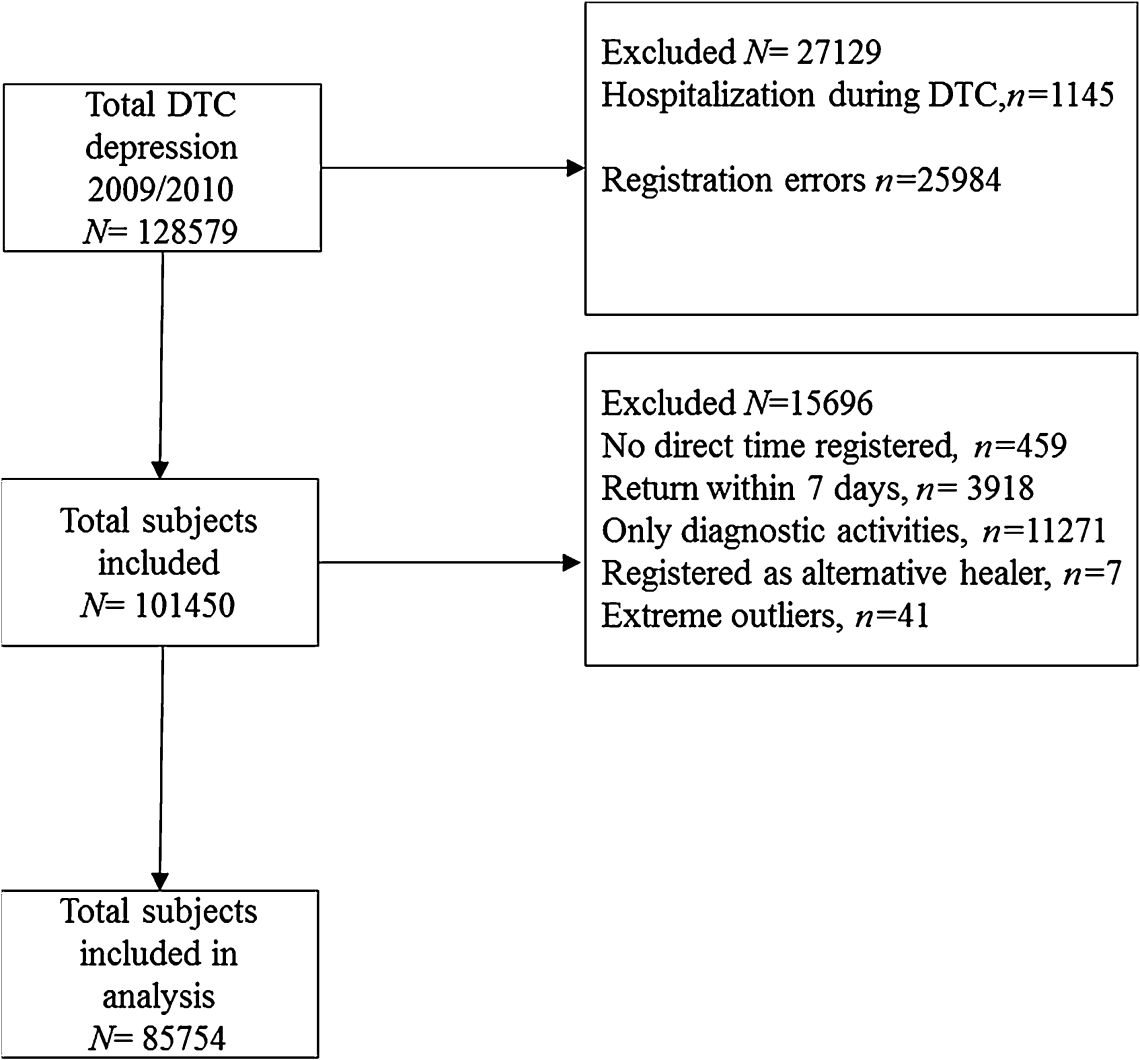
Subject flow chart

We excluded patients receiving other forms of treatment (for example only went through diagnostic procedures, or received inpatient treatment), patients for whom no treatment duration was registered, and patients without information on the termination of their treatment (e.g., if and when their treatment had finished). Furthermore, we excluded patients who were discharged from treatment, but returned within 7 days after discharge. We hypothesize that in those cases the treatment was probably ended for administrative reasons or registration inconsistencies (Fig. 1).

### Characteristics of treatment

#### Duration of treatment

Duration of the treatment episode was defined as the total time spent on face-to-face contacts in minutes. We categorized this time in: 5–250 min, 251–750 min, 751–1000 min and >1000 min. The treatment minutes were spent on the following activities:

- Diagnostic activities (intake, psychiatric/psychological/neurological/personality examination, intelligence testing, anamnestic interview).
- Psychological therapy (including all types of psychotherapy, counseling, psycho-education and other psychological activities).
- Pharmacotherapy (time spent on consultations).

Furthermore, information was available involving the termination of the treatment (terminated one-sided by client or therapist; terminated in collaboration). Information about number of sessions was not available due to registration inconsistencies (some practitioners registered every treatment session separately while others only register the total treatment time as one session).

There was no specified information available on the background and/or disciplines of the practitioners who performed treatment. However, all practitioners are licensed to work with patients in specialized mental health care. They are licensed and qualified to classify diagnoses and provide evidence-based treatments. The majority of patients were treated in large mental health centers (77.3 %, *n* = 66,293), while 22.7 % (*n* = 19,461) received treatment from self-employment psychiatrists or psychologists (e.g., professionals with own practice).

#### Return into mental health care

Return into mental health care was defined as: a new treatment episode within the same mental health provider after ending the initial treatment. It was not possible to identify patients who returned for care but switched to another provider. Data on return was available until December 2013, with exception of people who returned in 2013 and continued treatment in 2014. DTC data of 2014 was not yet available.

### Characteristics of the sample

#### Demographic variables

Gender and age were the only demographic variables available.

#### Clinical information

There was information available of comorbidity on all the DSM-IV-TR Axes (I–IV) [27]. For each of the axes comorbidity was coded as either present or absent.

Severity of complaints was assessed with the Global Assessment of Functioning (GAF) scale [27]. The GAF scores were registered at the start and end of the treatment. The GAF score ranges from 1 to 100, where 100 indicates no or few impairments in daily functioning, and 1 categorizes severe impairment in daily functioning. We categorized the GAF score into: 1–50; 51–100.

### Statistical analyses

All analyses were performed using SPSS 20 and a two sided significance level of *p* < 0.05 was used in all analyses. However, since multiple analyses were performed, we used a Bonferroni correction on the results [28]. We used the Holm–Bonferroni correction calculator (Excel) developed by Gaetano (2013) (results available on request).

First, multiple logistic regression analysis was performed to determine the association between the predictors and longer duration of treatment (>750 min). The cut-off point of 750 min in this specific analysis was based on the median. We conducted a series of univariate analyses for each predictor separately. Then, a backward multivariate analysis was performed. The assumptions of a logistic regression analyses were tested (linearity of the logit, multicollinearity and residual check) and there was no indication of any abnormality.

Second, a Cox regression model was used to test the relation between the predictors variables and the primary outcome, namely return into mental health care (0 = no return; 1 = return). We conducted a series of univariate analyses for each predictor separately. Then, a multivariate analysis was performed to determine the effect of individual predictors controlled for each other. Demographic variables (gender and age), clinical variables (comorbidity according to the DSM-IV-TR Axis, GAF score) and reason of finishing the treatment were first entered in the Cox regression analysis. Next, total time spent on face-to-face contacts was entered. The hazard ratio (HR) is the increase (or decrease) in risk of return into mental health care. A Cox regression adjusts for the different observation lengths of the data.

After the main analyses we examined whether the results were influenced by the received treatment. We divided the patients in four treatment categories: (1) patients who received psychotherapy only (e.g., psychoanalytic therapy, psychodynamic therapy, behavioral therapy, cognitive behavioral therapy, inter personal therapy, client focused therapy, systemic therapy, supportive therapy), (2) medication only (pharmacotherapy), (3) psychotherapy and medication, and (4) other therapy (e.g., skills training, creative therapy, psycho-education).

## Results

### Characteristics study sample

The study sample consisted of 64 % women (*n* = 54,716) (Table 1). The mean age of the sample was 44 (SD = 13.2) years. Diagnostic activities were registered for the majority of the sample (91 %). Supportive therapy was the most frequently registered treatment for the patients (44 %). At the beginning of treatment clients scored on average 60 (SD = 9.5) on the GAF and at the end of treatment 67 (SD = 12.3).

**Table 1:** Demographic and treatment characteristics of the study population and return into mental health care

	Overall population (*N* = 85,754) (100 %)		No return into mental health care (*n* = 73,677) (85.9 %)		Return into mental health care (*n* = 12,077) (14.1 %)		*χ* ^2^	*p*
	*N*	%	*N*	%	*N*	%		
Demographics								
Gender, women	54,716	63.8	46,999	63.8	7717	63.9	0.05	0.82
Age, mean (SD)^a^	44.1 (13.2)		44.2 (15.6)		43.5 (15.5)			
Age							15.17	0.001*
18–45	48,288	56.3	41,291	56.0	6997	57.9		
46–65	29,375	34.3	25,398	34.5	3977	32.9		
>65	8091	9.4	6988	9.5	1103	9.1		
Characteristics health care received								
Duration in minutes							235.55	<0.001*
5–250	11,598	13.5	9633	13.1	1965	16.3		
251–500	18,239	21.3	15,442	21.0	2797	23.2		
501–750	13,754	16.0	11,823	16.0	1931	16.0		
751–1000	11,027	12.9	9350	12.7	1677	13.9		
>1000	31,136	36.3	27,429	37.2	3707	30.2		
Treatment received^b, c^								
Diagnostic activities	77,996	91.0	67,450	91.5	10,591	87.7	181.28	<0.001*
Psychotherapy								
Psycho-analytic therapy	149	0.2	136	0.2	13	0.1	3.54	0.06
Psycho-dynamic therapy	4574	5.3	3882	5.3	692	5.7	4.37	0.04
Behavioral therapy	1589	1.9	1402	1.9	187	1.5	7.17	0.007*
Cognitive behavioral therapy	18,174	21.2	16,184	22.0	1990	16.5	187.15	<0.001*
Interpersonal therapy	1674	2.0	147	2.0	207	1.7	4.16	0.04
Client focused therapy	3754	4.4	3364	4.6	390	3.2	44.28	<0.001*
Systemic therapy	2245	2.6	1962	2.7	283	2.3	4.16	0.04
Supportive therapy, counseling	37,388	43.6	31,854	43.2	5534	45.8	28.26	<0.001*
Other therapeutic interventions^d^	19,627	22.9	17,155	23.3	2472	20.5	46.61	<0.001*
Creative therapy^e^	18,120	21.1	14,735	20.0	3385	28.0	401.37	<0.001*
Pharmacotherapy	31,886	37.2	27,269	37.0	4617	38.2	6.59	0.01
Treatment setting								
Psychotherapy only	36,944	43.1	32190	43.7	4754	39.4	100.06	<0.001*
Medication only	10,789	12.6	9222	12.5	1567	13.0		
Psychotherapy and medication	21,097	24.6	18,047	24.5	3050	25.3		
Other therapy	16,924	19.7	14,218	19.3	2706	22.4		
Severity of complaints								
GAF scores, mean (SD)^f^								
Start treatment^g^	60.0 (9.4)		60.0 (9.4)		60.0 (9.7)		98.88	<0.001*
End treatment^h^	67.0 (12.3)		67.0 (12.3)		66.0 (12.2)		212.44	<0.001*
As I comorbidity, yes	23,287	27.2	21,485	29.2	1802	14.9	1063.68	<0.001*
As II comorbidity^i^, yes	8810	10.3	8048	10.9	762	6.3	239.62	<0.001*
As III comorbidity, yes	29,234	34.1	25,500	34.6	3734	30.9	62.96	<0.001*
As IV comorbidity, yes	73,170	85.3	62,998	85.5	10172	84.2	13.57	<0.001
Reason for closing treatment							8.69	0.003*
Drop out client	23,055	26.9	19,675	26.7	3380	28.0		
In collaboration	62,699	73.1	54,002	73.3	8697	72.0		

* Significant after Bonferroni correction

^a^Range (18−110)

^b^Treatment was not restricted to one form of therapy

^c^For 1 % of the people (n=579) it is unknown what kind of treatment they received

^d^Activities cannot be categorized within forms of psychotherapy, like for example psycho-education or skills training

^e^Forms of therapy like drama and music therapy and other forms like psycho motoric therapy

^f^GAF scores range from 1−100, where 100 indicates no or few impairments in daily functioning

^g^ *n* overall population = 85,233; *n* no return = 73,248; *n* return = 11,985

^h^ *n* overall population = 84,547; *n* no return = 72,634; n return = 11,913

^i^Include personality disorders and other conditions that may cause problems

### Return to mental health care

Of the total sample (*N* = 85,754), 86 % did not return into mental health care (*n* = 73,677) within the observed period. The majority of patients who returned into mental health care, did so within the first year (73 %) after ending the initial treatment (*n* = 8833). More than half (56 %) of those who returned were diagnosed with a depression again (*n* = 6714), 1 % with other mood disorders (*n* = 142) and 7 % with an anxiety disorder (*n* = 897). The remaining patients (36 %) were diagnosed with a variety of other disorders (*n* = 4324). The majority of all the returning patients (92 %) received outpatient treatment again (*n* = 11,138), while 7 % returned for a crisis consult (inpatient and outpatient) (*n* = 782) and 1 % for other reasons (*n* = 157).

### Duration of treatment and its association with demographics and clinical variables

For duration of treatment, both univariate and multivariate regression analyses showed significant odds ratios (OR) for gender, age, comorbid problems on the DSM-IV Axes and reason of termination. Patients who were female, were younger, had comorbidity on other DSM-IV-TR axes and patients who ended their treatment in collaboration were more likely to receive a longer treatment (>751 min) (Table 2).

**Table 2:** Results univariate logistic model and backward logistic regression multivariate model longer duration of treatment (>751 min)

	Univariate			Multivariate		
	OR	95 % CI	*p*	OR	95 % CI	*p*
Determinants						
Female (ref: male)						
Total group	1.08	1.05–1.12	<0.001*	1.08	1.05–1.11	<0.001*
Psychotherapy only	1.13	1.08–1.18	<0.001*	1.13	1.08–1.18	<0.001*
Medication only	1.24	1.14–1.34	<0.001*	1.23	1.14–1.34	<0.001*
Psychotherapy and medication	1.11	1.05–1.18	<0.001*	1.10	1.04–1.17	0.002*
Other therapy only	1.22	1.14–1.31	<0.001*	1.21	1.12–1.30	<0.001*
Age (ref: >65)						
Total group						
18–45	1.46	1.39–1.53	<0.001*	1.52	1.45–1.60	<0.001*
46–65	1.36	1.29–1.42	<0.001*	1.37	1.30–1.44	<0.001*
Psychotherapy only						
18–45	1.74	1.61–1.89	<0.001*	1.84	1.69–1.99	<0.001*
46–65	1.60	1.48–1.74	<0.001*	1.61	1.48–1.76	<0.001*
Medication only						
18–45	1.61	1.41–1.84	<0.001*	1.47	1.28–1.69	<0.001*
46–65	1.37	1.19–1.57	<0.001*	1.30	1.13–1.49	<0.001*
Psychotherapy and medication						
18–45	1.41	1.29–1.536	<0.001*	1.37	1.24–1.51	<0.001*
46–65	1.24	1.12–1.37	<0.001*	1.19	1.08–1.32	0.001*
Other therapy only						
18–45	1.42	1.26–1.60	<0.001*	1.51	1.32–1.71	<0.001*
46–65	1.28	1.13–1.46	<0.001*	1.34	1.17–1.52	<0.001*
GAF score 6–10^a^ (start) (ref: 1–5)						
Total group	1.02	0.98–1.05	0.32	–	–	–
Psychotherapy only	1.04	0.98–1.09	0.18	–	–	–
Medication only	1.33	1.22–1.45	<0.001*	1.34	1.23–1.47	<0.001*
Psychotherapy and medication	1.14	1.07–1.21	<0.001*	1.14	1.07–1.22	<0.001*
Other therapy only	1.33	1.23–1.44	<0.001*	1.32	1.21–1.43	<0.001*
Comorbidity Axis I (ref: no)						
Total group	1.54	1.49–1.59	<0.001*	1.59	1.54–1.64	<0.001*
Psychotherapy only	1.45	1.38–1.51	<0.001*	1.53	1.46–1.60	<0.001*
Medication only	1.95	1.79–2.13	<0.001*	1.85	1.70–2.03	<0.001*
Psychotherapy and medication	1.50	1.40–1.61	<0.001*	1.48	1.38–1.59	<0.001*
Other therapy only	1.57	1.46–1.69	<0.001*	1.64	1.51–1.79	<0.001*
Comorbidity Axis II (ref: no)						
Total group	1.18	1.13–1.23	0.03	1.33	1.27–1.39	<0.001*
Psychotherapy only	1.26	1.18–1.35	<0.001*	1.43	1.33–1.53	<0.001*
Medication only	0.86	0.75–0.97	0.02	–	–	–
Psychotherapy and medication	0.92	0.84–1.004	0.06	–	–	–
Other therapy only	1.16	1.03–1.30	0.02*	1.34	1.18–1.51	<0.001*
Comorbidity Axis III (ref: no)						
Total group	1.10	1.07–1.13	<0.001*	1.13	1.10–1.17	<0.001*
Psychotherapy only	1.06	1.02–1.11	0.008*	1.12	1.07–1.17	<0.001*
Medication only	1.07	0.99–1.16	0.09	–	–	–
Psychotherapy and medication	0.94	0.89–1.001	0.05	–	–	–
Other therapy only	1.08	1.001–1.16	0.05	1.14	1.06–1.24	<0.001*
Comorbidity Axis IV (ref: no)						
Total group	1.70	1.64–1.77	<0.001*	1.66	1.60–1.73	<0.001*
Psychotherapy only	1.53	1.44–1.62	<0.001*	1.50	1.41–1.60	<0.001*
Medication only	2.30	2.05–2.86	<0.001*	2.13	1.89–2.40	<0.001*
Psychotherapy and medication	1.91	1.76–2.07	<0.001*	1.84	1.69–1.99	<0.001*
Other therapy only	1.39	1.27–1.52	<0.001*	1.43	1.30–1.57	<0.001*
Treatment closed in collaboration (ref: drop out of treatment)						
Total group	1.65	1.60–1.71	<0.001*	1.79	1.73–1.84	<0.001*
Psychotherapy only	1.86	1.77–1.95	<0.001*	1.99	1.89–2.09	<0.001*
Medication only	1.03	0.95–1.11	0.54	1.13	1.04–1.23	0.004*
Psychotherapy and medication	1.63	1.52–1.74	<0.001*	1.71	1.60–1.83	<0.001*
Other therapy only	1.58	1.47–1.770	<0.001*	1.70	1.58–1.84	<0.001*

Model psychotherapy only: *χ* ^2^ = 1463.41, *p* < 0.0001, *df* = 8). Mode medication only: *χ* ^2^ = 509.73, *p* < 0.0001, *df* = 7). Model psychotherapy and medication: *χ* ^2^ = 631.53, *p* < 0.0001, *df* = 7). Model other therapy: *χ* ^2^ = 513.404, *p* < 0.0001, *df* = 8)

* Significant after Bonferroni correction

Since drop-out of treatment is associated with treatment duration, differences between those who dropped out and those who ended their treatment in collaboration were collected. Those who dropped out were significantly younger (age category 18–45: 63 %) than those who did not (age category 18–45: 54 % *p* = < 0.01). Of the clinical variables, the patients who dropped out of treatment had more comorbidity on Axis I (drop out: 30 %; collaboration: 26 %, *p* = <0.01) and lower GAF scores at the end of treatment (drop out: 62; collaboration: 69, *p* = <0.01).

We also repeated the regression analyses for the patients who ended their treatment in collaboration only. The association between treatment duration and demographic/clinical variables for this subgroup were almost similar to those of the whole group (results available on request).

#### Duration of different types of treatment and its association with demographics and clinical variables

The univariate and multivariate regression analyses showed similar results for gender, age, GAF score and comorbidity with Axis I on treatment duration for the four different treatment groups (Table 2). Furthermore, people with comorbid axis II or axis III disorders more often received a long treatment than patients without those comorbid disorders, but only for those patients who received psychotherapy or other therapy. There was no association between comorbid axis II and III disorders and long treatment duration for people who had received medication or both medication and psychotherapy. Patients with a high GAF score more often received a long treatment than patients with a low GAF score. However, this association was not observed for patients who received only psychotherapy (Table 2).

### Duration of treatment and its association with return into mental health care

As shown in Table 3 both univariate and multivariate Cox regression analyses showed significant HR for GAF score, comorbidity with DSM-IV I, II axes and closing reason of treatment on return into mental health care. Patients who were younger (18–45 years), had a lower GAF score at the beginning of treatment, without comorbidity, as well as patients who dropped out of the treatment were more likely to return into mental health care. Patients who received a treatment duration between 5–250, 251–500 and 751–1000 min were more likely to return compared with the group who received longer than 1000 min (reference category) (HR: 1.19), 251–500 min (HR: 1.11) and 751–1000 min (HR: 1.18). There was no effect for people who received 501–750 min of therapy.

**Table 3:** Hazard ratios of determinants of subsequent return into mental health care

	Univariate			Multivariate					
	HR	95 % CI	*p*	HR	95 % CI	*p*	HR	95 % CI	*p*
				Step 1			Step 2		
Determinants									
Female (ref: male)									
Total group	1.005	0.97–1.04	0.81	–	–	–	–	–	–
Psychotherapy only	1.04	0.98–1.11	0.16	–	–	–	–	–	–
Medication only	0.96	0.87–1.06	0.41	–	–	–	–	–	–
Psychotherapy and medication	0.99	0.93–1.07	0.92	–	–	–	–	–	–
Other therapy only	0.99	0.92–1.08	0.82	–	–	–	–	–	–
Age (ref: >65)									
Total group									
18–45	1.07	1.007–1.14	0.03	1.09	1.02–1.16	0.009	1.10	1.04–1.18	0.003*
46–65	1.00	0.94–1.07	0.94	1.007	0.94–1.07	0.84	1.02	0.95–1.09	0.65
Psychotherapy only									
18–45	0.96	0.86–1.06	0.22	–	–	–	–	–	–
46–65	0.94	0.82–1.02	0.40	–	–	–	–	–	–
Medication only									
18–45	1.20	1.09–1.43	0.04	1.23	1.03–1.47	0.03	1.23	1.03–1.47	0.03
46–65	1.11	0.93–1.33	0.26	1.12	0.93–1.34	0.23	1.12	0.93–1.34	0.23
Psychotherapy and medication									
18–45	1.29	1.14–1.46	<0.001*	1.23	1.08–1.40	0.002	1.23	1.08–1.40	0.002*
46–65	1.12	0.98–1.28	0.10	1.08	0.95–1.25	0.25	1.08	0.95–1.247	0.25
Other therapy only									
18–45	1.04	0.91–1.18	0.58	–	–	–	–	–	–
46–65	0.99	0.86–1.13	0.83	–	–	–	–	–	–
GAF score 6–10^a^ (start) (ref: 1–5)									
Total group	0.81	0.78–0.84	<0.001*	0.80	0.76–0.82	0.000	0.78	0.75–0.82	<0.001*
Psychotherapy only	0.82	0.77–0.88	<0.001*	0.78	0.73–0.84	0.000	0.78	0.73–0.84	<0.001*
Medication only	0.87	0.79–0.98	0.02	0.86	0.77–0.95	0.005	0.86	0.77–0.95	0.005*
Psychotherapy and medication	0.85	0.79–0.91	<0.001*	0.86	0.80–0.92	<0.001	0.86	0.80–0.92	<0.001*
Other therapy only	0.76	0.70–0.82	<0.001*	0.71	0.65–0.78	<0.001	0.73	0.67–0.80	<0.001*
Comorbidity Axis I (ref: no)									
Total group	0.45	0.43–0.47	<0.001*	0.41	0.39–0.43	<0.001	0.42	0.40–0.44	<0.001*
Psychotherapy only	0.46	0.42–0.50	<0.001*	0.42	0.39–0.45	<0.001	0.43	0.39–0.46	<0.001*
Medication only	0.46	0.40–0.53	<0.001*	0.43	0.37–0.50	<0.001	0.43	0.37–0.50	<0.001*
Psychotherapy and medication	0.52	0.47–0.57	<0.001*	0.46	0.42–0.50	<0.001	0.46	0.42–0.50	<0.001*
Other therapy only	0.36	0.32–0.41	<0.001*	0.35	0.31–0.39	<0.001	0.36	0.32–0.41	<0.001*
Comorbidity Axis II (ref: no)									
Total group	0.58	0.54–0.62	<0.001*	0.47	0.44–0.51	<0.001	0.47	0.44–0.51	<0.001*
Psychotherapy only	0.57	0.50–0.64	<0.001*	0.46	0.41–0.52	<0.001	0.46	0.41–0.52	<0.001*
Medication only	0.62	0.51–0.76	<0.001*	0.52	0.42–0.63	<0.001	0.52	0.42–0.63	<0.001*
Psychotherapy and medication	0.54	0.47–0.62	<0.001*	0.44	0.39–0.51	<0.001	0.44	0.39–0.51	<0.001*
Other therapy only	0.63	0.53–0.74	<0.001*	0.51	0.43–0.61	<0.001	0.52	0.44–0.61	<0.001*
Comorbidity Axis III (ref: no)									
Total group	0.86	0.83–0.97	0.001*	0.89	0.85–0.92	<0.001	0.89	0.86–0.93	0.02
Psychotherapy only	0.91	0.86–0.97	0.005*	0.91	0.86–0.97	0.005	0.92	0.86–0.98	0.007*
Medication only	0.81	0.73–0.90	<0.001*	0.86	0.77–0.95	<0.001	0.86	0.77–0.95	<0.001*
Psychotherapy and medication	0.78	0.73–0.85	0.001*	0.83	0.77–0.90	<0.001	0.83	0.77–0.90	<0.001*
Other therapy only	0.88	0.81–0.96	0.002*	0.0.91	0.83–0.99	<0.001	0.0.91	0.83–0.98	<0.001*
Comorbidity Axis IV (ref: no)									
Total group	0.92	0.88–1.06	0.37	–	–	–	–	–	–
Psychotherapy only	0.99	0.92–1.09	0.99	–	–	–	–	–	–
Medication only	0.86	0.75–0.98	0.02	–	–	–	–	–	–
Psychotherapy and medication	1.06	0.96–1.18	0.27	1.13	1.02–1.26	0.02	1.13	1.02–1.26	0.02
Other therapy only	0.79	0.72–0.86	<0.001*	0.85	0.77–0.93	0.02	0.87	0.79–0.95	0.003*
Treatment closed in collaboration (ref: drop out of treatment)									
Total group	0.94	0.91–0.98	0.004*	0.93	0.89–0.96	<0.001	0.94	0.90–0.98	<0.001*
Psychotherapy only	0.96	0.90–1.03	0.22	–	–	–	–	–	–
Medication only	1.05	0.94–1.16	0.40	–	.	–	–	–	–
Psychotherapy and medication	0.83	0.77–0.90	<0.001*	0.83	0.77–0.90	<0.001	0.83	0.77–0.90	<0.001*
Other therapy only	1.04	0.96–0.1.13	0.35	–	–	–	–	–	–
Duration treatment (ref: >1000 min)									
Total group									
5–250 min	1.34	1.27–1.41	<0.001*				1.19	1.13–1.26	<0.001*
251–500 min	1.21	1.16–1.28	<0.001*				1.11	1.06–1.17	<0.001*
501–750 min	1.11	1.05–1.18	<0.001*				1.04	0.99–1.10	0.14
751–1000 min	1.24	1.17–1.31	<0.001*				1.18	1.11–1.25	<0.001*
Psychotherapy only									
5–250 min	1.43	1.30–1.57	<0.001*				1.26	1.15–1.39	<0.001*
251–500 min	1.25	1.15–1.35	<0.001*				1.14	1.05–1.24	0.001
501–750 min	1.09	1.09–1.19	0.05				1.006	0.92–1.10	0.89
751–1000 min	1.33	1.33–1.45	<0.001*				1.24	1.14–1.36	<0.001*
Medication only									
5–250 min	1.14	0.99–1.31	0.08				1.02	0.88–1.18	0.75
251–500 min	1.13	0.98–1.30	0.10				1.00	0.87–1.15	0.99
501–750 min	1.30	1.11–1.51	0.001*				1.21	1.04–1.41	0.02
751–1000 min	1.16	0.98–1.38	0.09				1.12	0.94–1.33	0.20
Psychotherapy and medication									
5–250 min	0.87	0.73–1.04	0.12				0.81	0.68–0.96	0.02
251–500 min	1.11	0.99–1.23	0.07				1.04	0.93–1.16	0.47
501–750 min	1.06	0.95–1.18	0.31				1.004	0.90–1.12	0.95
751–1000 min	1.32	1.19–1.45	<0.001*				1.26	1.13–1.39	<0.001*
Other therapy only									
5–250 min	1.90	1.68–2.16	<0.001*				1.67	1.47–1.89	<0.001*
251–500 min	1.66	1.47–1.88	<0.001*				1.53	1.34–1.73	<0.001*
501–750 min	1.50	1.30–1.72	<0.001*				1.43	1.24–1.64	<0.001*
751–1000 min	1.27	1.07–1.50	0.006*				1.26	1.06–1.49	0.008*

*HR* hazard ration, *CI* confidence interval

* Significant after Bonferroni correction

^a^GAF scores range from 1 to 100, where 100 indicates no or few impairments in daily functioning

#### Sensitivity analyses

To determine if the results from the Cox regression analyses were moderated by severity, an interaction term (comorbidity As I × total direct time, comorbidity As II × total direct time) was added to the model. The interaction terms were non-significant. Furthermore, additional analyses on return into mental health care were performed without the patients who dropped out of treatment. The additional Cox regression analyses showed that the effect of age was no longer significant in the multivariate analyses (HR 18–45 year = 1.03; 95 % CI 0.96–1.11; *p* = 0.42; HR 46–65 year = 0.94; 95 % CI 0.88–1.009; *p* = 0.08; >65 ref) but the other determinants remained almost similar (results available on request).

#### Duration of different treatments and its association with return into mental health care

Univariate and multivariate regression analyses showed similar results for gender, age, GAF score and comorbidity with Axis I and II on the risk to return into treatment irrespective of type of initial treatment. Furthermore, the Cox regression analyses showed a significant HR between return into treatment and patients who dropped out of treatment in the group who received psychotherapy and medication. This association was not found in the other treatment groups. Further, comorbidity with Axes IV was associated with a higher risk on return in the group who received other therapy (Table 3) but not for the other treatment groups.

The effect of duration of treatment and return into mental health care was similar for the group who received only psychotherapy and the group who received other therapy (compared to the total group) (Table 3). There was no effect of duration of treatment and return into mental health care for the people who received medication only and for people who received psychotherapy and medication. Sensitivity analyses were performed on all four groups separately. The interaction terms (comorbidity Axis I × total direct time, comorbidity Axis II × total direct time) were not significant for all four groups.

## Discussion

The aim of this study was to investigate whether duration of treatment and return into mental health care are related. A large dataset with naturalistic data from mental health care providers in the Netherlands was used. The results showed that the majority of patients (86 %) did not return into mental health care within the observation period. Patients who received a shorter duration of treatment were more likely to return than patients who received a longer duration of treatment (HR 5–205 min: 1.19; HR 251–500 min: 1.11; HR 751–1000 min: 1.18). The effect of duration of treatment on return into mental health care was different for patients who received medication only and patients who received both psychotherapy and medication.

Based on the population characteristics, the sample was not fully comparable with other cohorts [29, 30]. The comorbidity rates with other DSM Axis were relative low in our study (Axis I: 27 %, Axis II: 10 %) [29, 30]. This is most likely due to the fact that practitioners are instructed to report comorbidity only when it influences the treatment of the primary diagnose [26].

The most registered treatment in this sample was supportive therapy (44 %). This seems striking since guidelines indicate that Cognitive Behavioral Therapy (CBT), Cognitive Therapy (CT) and Interpersonal Therapy (IPT) are the preferred treatments in depression [2, 31]. However, it is common in mental health practice that practitioners work eclectically [31], possibly resulting in the use of relevant modules of the CBT protocol or other therapies and therefore register the treatment as supportive therapy. In addition, some psychiatrists report their consultations concerning medication follow-ups as supportive therapy; this may also explain the high percentage.

The data on return into mental health care showed that the majority of patients did not return (86 %). This percentage seems rather high, especially because several studies show that relapse rates for depression are high [18, 32], leading to frequent mental health care use. A positive explanation is that the treatments were effective for a majority of patients. Other explanations are that patients returned after our observation period, that they switched provider, or sought help in primary care or other settings. All this might have led to an underestimation of return into mental health care. Unfortunately, there was no possibility to examine underestimations of return in the present data sample. However, comparable data from other registries showed that about a quarter (26 %) of patients with a depression diagnosis switch provider when they return for depression to mental health providers (data not presented).

There is an increasing pressure on mental health-care systems to decrease the length of treatment, while retaining effectiveness [1]. While there is evidence that low intensity and brief treatments are effective [2–11], there is a lack of knowledge on the long-term outcome. The data in our study showed that patients who received a treatment duration of shorter than 500 min and 751–1000 min, were more likely to return compared with patients who received more than 1000 min, even when controlled for severity of symptoms. However, there is no clear line in the results, meaning that the effect of 5–250 min is similar to the effect of 751–1000 min. In addition there is no effect for the group who received 501–750 min. For patients who received other therapy (e.g., creative therapy, psycho-education), there was an association between all the different durations of treatment (5–250 min, 251–500 min, 501–750 min and 751–1000 min) and return into mental health care. However, similar to the results in patients who received psychotherapy, there is no clear line in the results, meaning that the effects of 5–250, 251–500, 501–750 and 751–1000 min are similar. In patients who received medication, no association between duration of treatment and return in mental health care was found.

A possible explanation for the differences between the groups is the purpose of the treatment. For example, multidisciplinary guidelines recommend that people who use pharmacotherapy return to their practitioner once in a while to examine the effect and/or stabilization of the provided medication [31]. Possibly, after a while, pharmacotherapy does not require the patient to be seen frequently in mental health care providers and they will probably only return for maintenance appointments. In contrast, psychotherapy and other forms of therapy require multiple face-to-face contacts and are often more frequently provided.

The similar HR found for different treatment categories (5–250 min, 251–500 min, 501–750 min and 751–1000 min) in the total group, patients who received psychotherapy only and patients who received other therapy is in contrast with other findings in naturalistic treatment settings. Those findings showed that treatment allocation is associated with severity of symptoms [15]; patients with more severe complaints received the most prolonged treatment [15]. Based on these results, we might expect that patients who received a longer treatment duration would be more likely to return into mental health care, due to the complexity of symptoms. One possible explanation is that patients who received a shorter treatment did not receive enough sessions to fully recover [13] or that their treatment was terminated too early, and that a longer duration of treatment might protect from return into mental health care. Another explanation is that the patients who dropped out of treatment (and consequently received a shorter duration) were at higher risk for return due to the complexity of symptoms. However, the results of the sensitivity analyses (excluding patients who dropped out of treatment) were comparable on almost all determinants. A third explanation might be that some patients have a more frequent pattern of health care use, where they receive a shorter initial duration of treatment but return more often for one or two booster sessions. In line with this idea, the multidisciplinary guidelines for depression emphasize the importance of relapse prevention [31]. Unfortunately, it is not possible to test this hypothesis in the present study.

Based on the findings of our study, at least some patients might benefit more from a longer duration of treatment. However, an important consideration is that the effects found for all the analyses were relative small. Furthermore, population characteristics showed that only a minority of patients received treatment between 5–250 min (14 %) and between 251–500 min (21 %). Therefore, and because of the naturalistic design of the study, it is not possible to draw causal conclusions about the use of brief therapies en the risk of relapse into repeated mental health care use. However, our findings do suggest that brief therapies may not be sufficient for every patient when we look at the long-term outcome. Further research, preferably in a controlled setting of a RCT, should examine if brief therapies are associated with repeated mental health care use.

This study has several strengths and limitations that need to be considered. An important strength of the study is that we examined a naturalistic cohort. Furthermore, it was a large sample and included specialized mental health providers in the Netherlands. A disadvantage of this design is that the registration system was not specifically designed for scientific research purposes. As a consequence, we had no control over the registration of the data (e.g., there was no possibility to check registration errors or misclassifications) which may have influenced the results. However, the reliability of the data in the registration is tested by the insurance companies periodically in order to verify the claims made. This is no guarantee of the reliability of the data, but does ensure that much effort goes into the quality of the data that are stored in the DTC information. We examined the association of brief treatment and return into mental health care, but had no control over who received brief or prolonged treatment. Variability may exist in how practitioners will organize and close the treatment, especially for patients with severe symptoms [15]. In addition, we followed every individual patient without additional background information (e.g., history of depression and previous help-seeking behavior). Finally, with the data available to us at the time of the analysis, the number of practitioners and institutions was unknown. Therefore, it was not possible to control for the potentially nested structure of the data.

## Conclusion

The findings of this study provide some indication that patients who receive a longer duration of treatment may be less likely to return into mental health care. However, further research, preferably using controlled and randomized designs, is needed to determine if the trend towards lower intensive and brief treatment is associated with more relapse and return into mental health care.
